# Congenital Insensitivity to Pain without Anhidrosis: Orodental Problems and Management

**DOI:** 10.1155/2015/179892

**Published:** 2015-09-20

**Authors:** N. Abdullah, Kausar Sadia Fakhruddin, A. R. Samsudin

**Affiliations:** College of Dental Medicine, University of Sharjah, UAE

## Abstract

This paper reports the case of a 4-year-old male patient who was brought by parents requesting for replacement of multiple missing anterior teeth. The patient suffered from congenital insensitivity to pain without anhidrosis and presented with full blown sequelae of the condition in the form of oral self-mutilation leading to loss of teeth, tongue tip amputation, finger tips destruction, and lower limb wound infections. Dental and orthopaedic treatment consists of local management of oral wound and prevention from further oral and finger injuries that takes the form of dental splints and finger sleeve splints, constant feet coverage with shoes, and behavioural medical therapy. The age of the patient and parents' education present challenges in managing this condition to avoid morbidity and premature mortality.

## 1. Introduction

Pain is a common unpleasant experience often caused by intense or damaging stimuli. Acquired insensitivity to pain such as diabetic neuropathy is a common condition. However, congenital insensitivity to pain with or without anhidrosis [1–3] is a rare condition and is often diagnosed in the late toddler age when the child is more active and prone to painless falls [4, 5]. Despite protection from potential external harmful insults, the patient themselves self-mutilate their own body, chewing their own tongue, lips, and fingers destructively [4–8].

These cases are very often referred to dental clinics for emergency management of acute oral trauma resulting from their neurological condition. This case report describes a case of a four-year-old boy with congenital insensitivity to pain that was brought by his parents seeking dental help for prostheses to replace his self-extracted primary teeth at the University Dental Hospital Sharjah, United Arab Emirates.

## 2. Case Presentation

A four-year-old Arab-Egyptian boy was brought by parents to pediatric clinic seeking prosthetic replacement of multiple missing teeth. The patient was elder of two siblings from a consanguineous marriage. History revealed the patient was born at full term through normal delivery and both parents and the younger brother were healthy.

**Figure 1:**
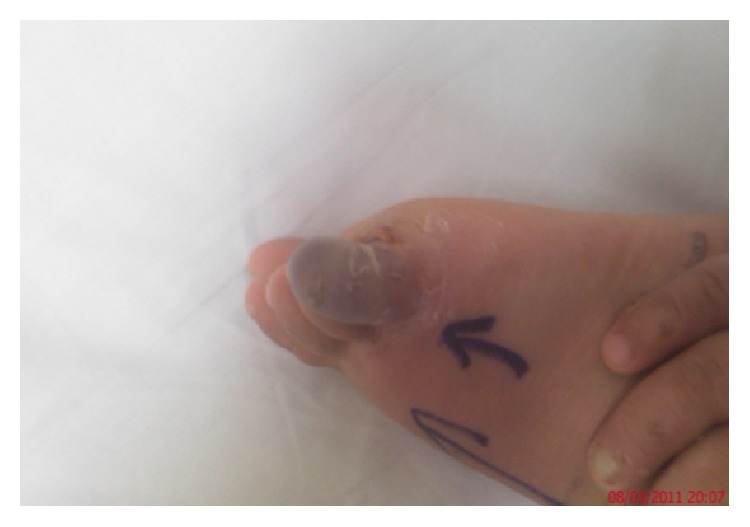
Photograph of the right foot showing necrosed right little toe resulting from severe infection before amputation at age of 2.8 years.

Past medical history showed previous hospital admission at the age of two years and ten months for emergency intervention to treat chronic osteomyelitis and periostitis that resulted in swollen right foot above the talus. The condition at that time was diagnosed as septic arthritis and acute osteomyelitis. The condition in the right foot recovered well albeit with some scarring. At age three, patient's right little toe got hurt while playing for which he obviously never complained, which later got infected leading to necroses and ultimate amputation (Figure 1). At this stage he was diagnosed as suffering from congenital insensitivity to pain without signs of anhidrosis. The pediatric neurologist confirmed his poor attention and concentration span and a pediatric orthopedic surgeon diagnosed his right foot condition as “charcot arthropathy.” He sleeps well at night and sweat profusely particularly when the room is warm.

**Figure 2:**
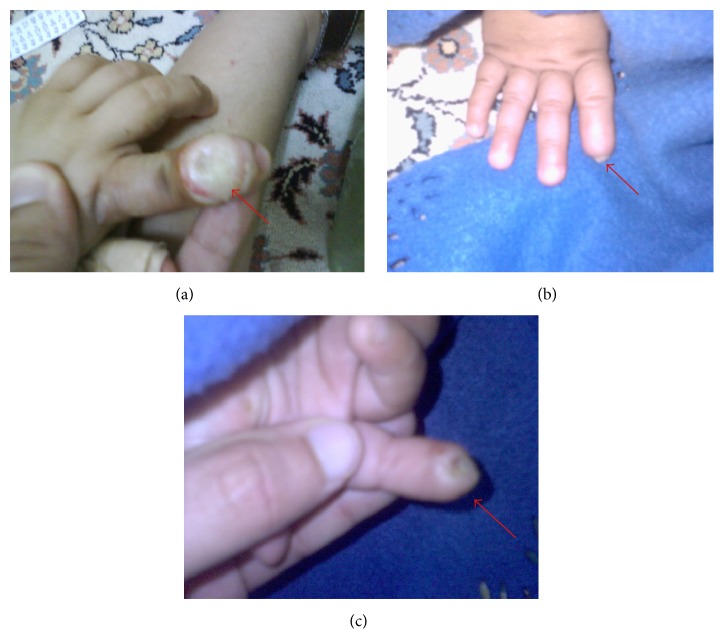
(a) Photograph at age two, showing self-inflicted traumatic ulcer due to biting. (b) Photograph at age four, showing self-amputation of the terminal phalanges of the right index finger from dorsal aspect. (c) Photograph at age four, showing self-amputation of the terminal phalanges of the right index finger from ventral aspect.

The parents noticed that the patient is suffering from some chewing difficulties due to premature tooth loss but without swallowing difficulty and this was the main reason that had brought them to seek dental treatment. General examination showed a fully alert patient with hyperactive behavior and short attention span. The patient's height and weight was appropriate for his age with normal gait and posture. There was an extensive damage to index finger of the right hand due to biting and chewing (Figures 2(a), 2(b), and 2(c)).

**Figure 3:**
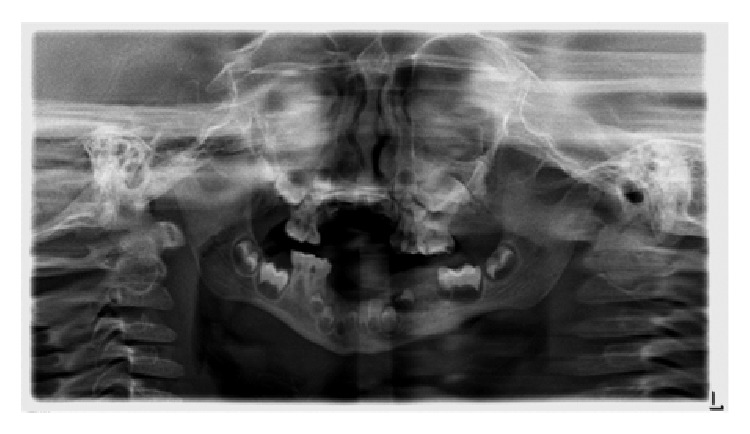
An orthopantomogram (OPG) taken at age of four, showing the maxilla and mandible with few standing deciduous teeth and maturation stage of permanent maxillary and mandibular teeth.

Clinical oral examination demonstrated scars on tip of the tongue and around vermillion border of the lower lip suggesting self-inflicting injuries probably due to pain insensitivity. The tongue was of normal size and color but bald and depapillated. Intraoral hard-tissue examination demonstrates no carious lesion. There was an open wound in the left mandibular quadrant with exposed alveolar bone due to a very recent self-extraction of two deciduous molar teeth (74, 75), according to the history. Distal to the wound, there was premature exposure of mesiobuccal cusp tips of the left mandibular first permanent molar. In addition, severely resorbed maxillary and mandibular alveolar ridges were evident clinically and radiographically. The orthopantomogram (OPG) examination of the jaw bones showed normal jaw anatomy and bone density (Figure 3). The crowns of permanent teeth development are consistent with the physiological and chronological age of the patient. However, both mandibular permanent second premolars are absent.

It was noted that piercing the buccal gingiva with a sharp dental probe during oral examination did not elicit any physical reaction from the child.

Based on the available pediatric, orthopedic, and neurological records, a diagnosis of oral self-mutilation secondary to congenital insensitivity to pain was established.

## 3. Discussion

In this case, a young child who is devoid of normal pain sensation suffers injuries in several parts of the body without showing any disability due to pain. Mardy and his colleagues [9] reported the role of consanguineous marriage resulting in this condition. Both parents are asymptomatic and gave a history of consanguineous marriage. A genetic mutation related to this disorder has been identified and classified as Hereditary Sensory and Autonomic Neuropathy (HSAN) type III [2].

The destructive behaviour of self-tooth extraction and other oral manifestations have been reported by many centers managing these special children [4–8]. This oral habit could have started with nail and later finger biting during the episodes of eruption of deciduous anterior and the abnormal feelings made the child do it more. The deciduous incisors later become loose and were easily avulsed by the child.

The poorly healed lower left second deciduous molar extraction socket with exposed alveolar bone is likely due to continuous trauma on the painless healing granulation tissue giving rise to areas of mucosal necrosis and sequestrated superficial alveolus. Continuous traumatic contact between the upper and lower dentoalveolar region could have led to severe resorption of the maxillary and mandibular alveolar ridges which may be responsible for premature exposure of the mesiobuccal cusp tips of the left mandibular first permanent molar.

These children may be shown photographs of lip injury to educate them not to bite their own lips. However, these habits may perpetuate during sleep as part of a sleep disordered syndrome in these children. In such circumstances, soft vacuum dental splints or hard acrylic splints may be used to cap either mandibular or maxillary teeth or both in an attempt to break the lip and tongue biting habit. This is only possible when the deciduous molars have erupted and able to retain the splints. Littlewood and Hutton [10, 11] recommended using soft occlusal guards to prevent dental self-mutilating injuries in their cases together with behavior management techniques.

The poorly healing wound in the patient's mouth was managed by using local measures that include wound debridement and mouth rinsing with chlorhexidine 0.12% solution. In this case provision of prostheses was not possible due to deficient tooth-tissue support, from severely resorbed upper and lower dentoalveolar ridges resulting from self-extractions. In addition, implant retained prostheses were not possible due to presence of unerupted teeth contraindicating implant placement.

Preventive measures such as custom fitted shoes, education regarding foot care, changing posture to relieve continuous pressure on bones and joints can prevent the need of multiple surgeries for injuries [12]. Medical treatment such as using amphetamines or dextroamphetamine [13, 14] for behavioral control of hyperactivity perhaps can further contribute to protect these children from physical injuries.

A multidisciplinary team approach comprising the neurologist, psychologist, family physician, and pediatric dentist from whom the child needs specialized care shall collectively provide the holistic care approach. In addition, we need to educate both the child and parents to ensure that the child is always in a safe environment.

## References

[1] Hilz M. J., Axelrod F. B., Bickel A. (2004). Assessing function and pathology in familial dysautonomia: assessment of temperature perception, sweating and cutaneous innervation. *Brain*.

[2] Nagasako E. M., Oaklander A. L., Dworkin R. H. (2003). Congenital insensitivity to pain: an update. *Pain*.

[3] Axelrod F. B., Hilz M. J., Vinken P. J., Bruyn G. W. (2000). Familial dysautonomia. *Handbook of Clinical Neurology*.

[4] Erdem T. L., Ozcan I., Ilgüy D., Sirin S. (2000). Hereditary sensory and autonomic neuropathy: review and a case report with dental implications. *Journal of Oral Rehabilitation*.

[5] Narayanan V. (1996). Oral and maxillofacial manifestations of hereditary sensory neuropathy. *British Journal of Oral and Maxillofacial Surgery*.

[6] Amano A., Akiyama S., Ikeda M., Morisaki I. (1998). Oral manifestations of hereditary sensory and autonomic neuropathy type IV. Congenital insensitivity to pain with anhidrosis. *Oral Surgery, Oral Medicine, Oral Pathology, Oral Radiology, and Endodontics*.

[7] Bodner L., Woldenberg Y., Pinsk V., Levy J. (2002). Orofacial manifestations of congenital insensitivity to pain with anhidrosis: a report of 24 cases. *Journal of Dentistry for Children*.

[8] Neves B. G., Roza R. T., Castro G. F. (2009). Traumatic lesions from congenital insensitivity to pain with anhidrosis in a pediatric patient: dental management. *Dental Traumatology*.

[9] Mardy S., Miura Y., Endo F., Matsuda I., Indo Y. (2001). Congenital insensitivity to pain with anhidrosis (CIPA): effect of TRKA (NTRK1) missense mutations on autophosphorylation of the receptor tyrosine kinase for nerve growth factor. *Human Molecular Genetics*.

[10] Littlewood S. J., Mitchell L. (1998). The dental problems and management of a patient suffering from congenital insensitivity to pain. *International Journal of Paediatric Dentistry*.

[11] Hutton A., McKaig S. (2010). The dental management of a child with congenital insensitivity to pain. *Dental Update*.

[12] Bar-On E., Weigl D., Parvari R., Katz K., Weitz R., Steinberg T. (2002). Congenital insensitivity to pain—orthopaedic manifestations. *The Journal of Bone and Joint Surgery—British Volume*.

[13] Faraone S. V., Biederman J. (2002). Efficacy of adderall for attention-deficit/hyperactivity disorder: a meta-analysis. *Journal of Attention Disorders*.

[14] http://www.fda.gov/Drugs/DrugSafety/PostmarketDrugSafetyInformationforPatientsandProviders/ucm107918.htm.

